# Hold the Nitro: Acetylcholine Provocation Testing for Coronary Vasospasm

**DOI:** 10.1016/j.jscai.2025.103760

**Published:** 2025-07-23

**Authors:** Adam S. Vohra, Eric A. Osborn

**Affiliations:** a Section of Cardiology, Department of Medicine, The University of Chicago, Chicago, Illinois; b Division of Cardiology, Beth Israel Deaconess Medical Center, Harvard Medical School, Boston, Massachusetts

**Keywords:** acetylcholine provocation, angina with nonobstructive coronary artery disease, coronary artery vasospasm, coronary microvascular dysfunction

Coronary artery vasospasm (CAS) is a common, but often underrecognized, cause of angina with nonobstructive coronary artery disease (ANOCA).^1^ Although intracoronary acetylcholine (Ach) provocation testing was first described in the 1980s to diagnose CAS,^2^ only in recent years have both American and European guidelines recommended invasive diagnostic coronary function testing for patients with ANOCA.^3^^,^^4^ Despite these recommendations, there remains significant variability in the clinical practice patterns for CAS testing with ongoing uncertainty as to how the timing of Ach provocation testing may impact diagnostic results. Although some centers prefer Ach provocation prior to coronary microvascular dysfunction (CMD) testing—which may involve nitroglycerin administration—others worry that induction of spasm may affect coronary microvascular resistance and coronary flow and, therefore, perform Ach after coronary thermodilution measurements.^5^

In this issue of *JSCAI*, Rehan et al^6^ evaluate the effect of nitroglycerin on the reproducibility of Ach provocation. In their analysis of 40 patients with epicardial CAS positive by invasive Ach testing, they found that when Ach provocation rechallenge was performed after intraarterial nitroglycerin administration, only 55% of patients redemonstrated epicardial spasm after 10 minutes. Of note, an additional 15% of patients demonstrated only microvascular spasm, which was defined as clinical symptoms and ischemic electrocardiogram changes during Ach provocation in the absence of >90% angiographic coronary artery diameter reduction. This raises concerns regarding the sensitivity of Ach provocation after nitroglycerin administration, which is often given not only intracoronary during CMD testing but also intraarterially after radial artery access.

Although it remains unclear whether higher dose Ach and longer time intervals after nitroglycerin administration affect CAS diagnostic yield, the authors should be commended for providing useful insights that may alter clinical workflows for patients undergoing invasive testing for ANOCA. Furthermore, the study raises questions about whether, in addition to under testing for CAS in patients with ANOCA, there may also be underdiagnosis due to certain testing protocols. The results are similar to prior work by Seitz et al^7^ demonstrating that Ach rechallenge after intracoronary nitroglycerin administration was highly effective in preventing epicardial spasm, but in cases of coexisting epicardial and microvascular spasm, could unmask the microvascular component.

Despite the important data provided in the study, the nature of the study leaves many questions unanswered. First, although best practice is to avoid the use of nitroglycerin prior to CAS testing, there may be cases, such as wire-based or radial artery spasms, where its use may be necessary. Although the motivation for this study stemmed from the timing of Ach provocation in relation to CMD testing, the timing of rechallenge was in relation to intraarterial nitroglycerin in the study. Subjects enrolled not only received intracoronary nitroglycerin after CMD testing but also were given additional intraarterial nitroglycerin prior to Ach rechallenge. This leaves lingering uncertainty regarding which is the major factor in decreasing CAS diagnostic yield. Second, the authors used a threshold of >90% reduction in coronary artery diameter to diagnose epicardial spasm, which is the standard in most guidelines and consensus statements^5^; however, 2-dimensional angiography interpretation is subjective and often variable when based on visual severity estimation and therefore may not fully capture the impact of CAS on coronary flow, opening potential opportunities to investigate invasive physiology measurements or newer angiography-derived indices as a future area of study. Finally, there may have been other factors that influenced CAS diagnostic yield, such as sedation, that were not controlled for.

More broadly though, this study highlights limitations for evaluating CAS when considering ad hoc testing for ANOCA, especially with the increase in radial artery access, which often utilizes intraarterial nitroglycerin administration.^8^ For patients with angina, nearly 50% have nonobstructive coronary artery disease^1^ and a smaller percentage of those patients receive comprehensive coronary function testing. In selected patients, ad hoc testing for CMD and CAS holds significant promise for securing a diagnosis at the time of cardiac catheterization and then initiating appropriate tailored medical therapy, avoiding the potential need to bring back patients for additional procedures.

It has been nearly 40 years since Ach provocation was first described; however, challenges remain for the diagnosis and treatment of CAS. This study, along with the others that preceded it, highlights difficulties in creating standardized protocols for CAS testing. Additionally, barriers to Ach usage also exist, because of the lack of published data on Ach stability after pharmacy compounding and intermittent national Ach supply chain shortages that can delay these procedures. Further research is needed to standardize and optimize invasive testing for ANOCA.
